# Extreme Beta-Cell Deficiency in Pancreata of Dogs with Canine Diabetes

**DOI:** 10.1371/journal.pone.0129809

**Published:** 2015-06-09

**Authors:** Emily J. Shields, Carol J. Lam, Aaron R. Cox, Matthew M. Rankin, Thomas J. Van Winkle, Rebecka S. Hess, Jake A. Kushner

**Affiliations:** 1 McNair Medical Institute, Pediatric Diabetes and Endocrinology, Baylor College of Medicine, Texas Children’s Hospital, Houston, TX, United States of America; 2 Pediatric Endocrinology and Diabetes, Children’s Hospital of Philadelphia, Perelman School of Medicine, University of Pennsylvania, Philadelphia, PA, United States of America; 3 Matthew J. Ryan Veterinary Hospital, School of Veterinary Medicine, University of Pennsylvania, Philadelphia, PA, United States of America; University of British Columbia, CANADA

## Abstract

The pathophysiology of canine diabetes remains poorly understood, in part due to enigmatic clinical features and the lack of detailed histopathology studies. Canine diabetes, similar to human type 1 diabetes, is frequently associated with diabetic ketoacidosis at onset or after insulin omission. However, notable differences exist. Whereas human type 1 diabetes often occurs in children, canine diabetes is typically described in middle age to elderly dogs. Many competing theories have been proposed regarding the underlying cause of canine diabetes, from pancreatic atrophy to chronic pancreatitis to autoimmune mediated β-cell destruction. It remains unclear to what extent β-cell loss contributes to canine diabetes, as precise quantifications of islet morphometry have not been performed. We used high-throughput microscopy and automated image processing to characterize islet histology in a large collection of pancreata of diabetic dogs. Diabetic pancreata displayed a profound reduction in β-cells and islet endocrine cells. Unlike humans, canine non-diabetic islets are largely comprised of β-cells. Very few β-cells remained in islets of diabetic dogs, even in pancreata from new onset cases. Similarly, total islet endocrine cell number was sharply reduced in diabetic dogs. No compensatory proliferation or lymphocyte infiltration was detected. The majority of pancreata had no evidence of pancreatitis. Thus, canine diabetes is associated with extreme β-cell deficiency in both new and longstanding disease. The β-cell predominant composition of canine islets and the near-total absence of β-cells in new onset elderly diabetic dogs strongly implies that similar to human type 1 diabetes, β-cell loss underlies the pathophysiology of canine diabetes.

## Introduction

Dogs develop naturally-occurring diabetes mellitus which is clinically similar to type I diabetes in humans. However, the utility of this large animal spontaneous disease model is limited by the fact that little is known of the etiology and pathophysiology of canine diabetes. Canine diabetes is one of the most common and devastating diseases of companion animals, affecting about 1 in 300 dogs [1–4]. Diabetes in dogs strongly resembles human type 1 diabetes mellitus (T1DM), with progressive onset of hyperglycemia, polydipsia, polyuria, ketonuria, diabetic ketoacidosis, and death without lifetime injected insulin therapy. There is no evidence for human type 2-like diabetes mellitus occurring in dogs (3). The vast majority of new onset cases of canine diabetes are in middle age or elderly dogs, with peak age of onset at age 9 [5, 6]. Several breeds are at higher risk for canine diabetes, including Samoyeds, Miniature Schnauzers, Miniature Poodles, Pugs, Toy Poodles, and Australian Terriers [2]. Notably, several other forms of canine diabetes have been described, including diabetes which develops secondary to diestrus and pregnancy and may be reversible with ovariohysterectomy congenital diabetes [7, 8], pancreatitis associated diabetes [9], and early-onset diabetes (less than a year of age) [4].

The pathophysiology of canine diabetes remains poorly understood [4, 10]. Various theories of the cause of canine diabetes have been proposed. Autoimmune etiology of canine diabetes is supported by genetic association of a major histocompatibility complex (MHC) haplotypes [11]. Because the MHC region is under strong linkage disequilibrium, it has not been thus far possible to definitively identify causative mutations in canine diabetes [12]. Similar to human diabetes, Gad65 and IA-2 antibodies have been associated with canine diabetes by some authors [13] but in a recent large and detailed study these antibodies were not identified [10]. Insulin gene polymorphisms have been associated with canine diabetes in various breeds [14]. CTLA4 promoter polymorphisms have also been associated with canine diabetes in various breeds [15]. Pancreatitis has also been suggested to be a significant contributor to the etiology of canine diabetes, especially in some breeds such as Miniature schnauzer [4]. Similarly, pancreatic exocrine disease and canine diabetes have been linked in German shepherds and Cocker spaniels [4]. A distinct neonatal form of canine diabetes has also been described in specific breeds including Labrador retrievers [16].

Surprisingly little is known about the histopathology of islet phenotypes in canine diabetes. The literature is limited to a few qualitative studies [10, 13, 17]. Other histopathology studies were performed in very young dogs with islet hypoplasia [7]. But, no detailed pancreatic morphometric study has been yet performed to conclusively survey the histopathology of the endocrine pancreas in canine diabetes. This is particularly notable given the longstanding speculation regarding the possibility of exocrine pancreas disease contributing to canine diabetes [4]. We hypothesized that novel insights could be gained into the pathophysiology of canine diabetes by performing a detailed survey of the histopathology of pancreata from diabetic dogs. Here we show that canine diabetes is associated with extreme β-cell loss characterized by a profound deficiency of β-cells within virtually all islets, without any evidence for pancreatitis or infectious etiology in most cases.

## Materials and Methods

### Sample Population

Our study population was comprised of dogs that were humanely euthanized and had a necropsy performed at the request of their owners at the Matthew J. Ryan Veterinary Hospital University of Pennsylvania School of Veterinary Medicine. Dogs were euthanized between the years of 2000 and 2012, prior to the onset of the present study, and for reasons unrelated to the study. Pancreata for the study were identified retrospectively from the archives of the necropsy service. Therefore, there was no need for Institutional Animal Care and Use Committee approval. The dog’s owners provided permission for their animal's tissues to be stored and used for research (whether or not a necropsy is performed). The consent form was signed by all owners of all pets at the time of admission. Analyses of islet endocrine/beta cell area, islet composition, proliferation, and insulin-glucagon coexpression were performed using a random subset of samples. Diabetic samples were chosen for CD3 analysis based on residual islet mass and availability of slides.

### Histopathology

17 control and 23 diabetic pancreas samples were obtained from the pathology archives. Veterinary care records were consulted to confirm diabetes mellitus and diabetic ketoacidosis. Slides stained with hematoxylin and eosin (H&E) were examined by a pathologist to confirm pancreatitis.

### Pancreas Morphometry

Slides were stained with H&E or Masson’s Trichrome stain by Children’s Hospital of Philadelphia pathology. Slides were scanned digitally via Aperio (Leica Biosystems, Buffalo Grove, IL).

### Immunohistochemistry

Pancreatic sections were rehydrated, permeabilized with 1% Triton X-100 in PBS, and microwaved in 0.01 M sodium citrate (pH 6.0) at 100% power for 8 min, 30% power for 20 minutes to retrieve antigens before primary antisera, as previously [18]. Primary antisera included guinea pig anti-insulin (Life Technologies, Grand Island, NY), rabbit anti-synaptophysin (ab10988, Life Technologies), mouse anti-ki67 (550609, BD Biosciences, San Jose, CA), rabbit anti-islet cocktail (rabbit anti-islet somatostatin and rabbit anti-pancreatic polypeptide; 18–0078 and 100–043, Life Technologies), mouse anti-glucagon (ab10988, Abcam) and rabbit anti-CD3 (PA1-37282 Life Technologies). Secondary antibodies were labeled with donkey anti-cy2, donkey anti-cy3, or donkey anti-cy5 (Jackson ImmunoResearch, West Grove, PA). Nuclear staining was performed with 4’6-diamidino-2-phenylindole (DAPI; Life Technologies). Slides were imaged to quantify β-cell and islet endocrine cell morphometry as previously described [18].

### Islet Morphometry

β-cell and islet endocrine cell cross-sectional area were measured by acquiring images on a Zeiss microscope (Zeiss, Thornwood, NY) using a 5x objective and a 0.63x converter and a Ludl X-Y motorized stage (Ludl, Hawthorne, NY), with antisera against insulin and synaptophysin, as previously [18]. Total pancreatic images were acquired by photographing pancreatic autofluorescence with GFP filters (Chroma, Bellows Falls, VT). Insulin and synaptophysin were imaged with Cy3 and Cy5, respectively. Images were analyzed for insulin-positive (β-cells) or synaptophysin-positive (islet endocrine cells) area with Volocity (PerkinElmer, Waltham, NY). Results of β-cell and islet endocrine cell quantification were expressed as the percentage of the total pancreas area surveyed containing islet cells positive for insulin or synaptophysin, respectively.

### Statistics

All results were reported as mean ± standard error of the mean (S.E.M). Results were compared with independent t-tests (unpaired and two-tailed) reported as P values.

## Results

### Severe hyperglycemia and diabetic ketoacidosis in dogs

The study population was comprised of diabetic and control dogs treated at the Matthew J. Ryan Veterinary Hospital. Most dogs were euthanized at the hospital, although 2 controls were dead on arrival (S1 Table). The 23 diabetic dogs spanned a wide age range at onset (from 3 months to 15.5 years old) with average of 8 years (Table 1), typical of published reports [2]. 17 control dogs were selected to match age and sex distributions of the diabetic cohort. Controls had been euthanized for a variety of causes. Intact males and females were present in equal distribution, as well as castrated males and spayed females of various breeds. Newly diagnosed and previously diagnosed diabetics were also included, with disease duration from new onset to longstanding disease, were used in the study. 11 dogs were recent onset diabetics (0–63 days duration). 10 dogs had diabetes for more than a year (up to nearly 10 years duration). Average disease duration was 431.3 days.

**Table 1:** Sample population.

	Animal	Age (days)	Age (years)	Sex	Castrated (Y/N)	Spayed (Y/N)	Breed	Weight (kg)	Blood Glucose (mg/dl)	Pancreatits (Y/N)	DKA (Y/N)	Diab. onset (years)	Diab. duration (days)
**Control**	cont. 1	4526	12.40	M	Y	-	Mixed Breed	5.7	113	N	N	-	-
	cont. 2	5083	13.93	F	-	Y	Mixed Breed	12.9	95	N	N	-	-
	cont. 3	4575	12.53	M	Y	-	English Springer Spaniel	21	78	N	N	-	-
	cont. 4	3449	9.45	M	Y	-	Golden Retriever	37.4	93	N	N	-	-
	cont. 5	508	1.39	F	-	N	Mixed Breed	7.94	95	N	N	-	-
	cont. 6	1730	4.74	F	-	Y	Cane Corso	42.4	107	N	N	-	-
	cont. 7	3823	10.47	M	Y	-	Basset Hound	25.8	138	N	N	-	-
	cont. 8	5295	14.51	F	-	Y	Mixed Breed	8	65	N	N	-	-
	cont. 9	4951	13.56	M	Y	-	Toy Poodle	3.5	27	N	N	-	-
	cont. 10	3123	8.56	M	Y	-	Welsh Springer Spaniel	23.9	106	N	N	-	-
	cont. 11	5338	14.62	M	Y	-	Dalmation	25	96	N	N	-	-
	cont. 12	3126	8.56	M	Y	-	Irish Terrier	30	59	N	N	-	-
	cont. 13	2913	7.98	F	-	Y	Greyhound	28.3	127	N	N	-	-
	cont. 14	4742	12.99	M	Y	-	Mixed Breed	4.3	91	N	N	-	-
	cont. 15	4491	12.30	M	Y	-	Belgian Malinosis	16	82	N	N	-	-
	cont. 16	3542	9.70	F	-	Y	Cairn Terrier	8.3	66	N	N	-	-
	cont. 17	3837	10.51	M	Y	-	Shetland Sheepdog	16.6	170	N	N	-	-
	average	3826.6	10.48	65%	100%	83%		18.6	94.6	0.0	0.0		
	std error	322.6	0.88					2.9	8.0				
**Diabetic**	diab. 1	3923	10.75	M	Y	-	Pomeranean	6.8	450	N	N	10.25	182
	diab. 2	2344	6.42	F	-	Y	Toy Fox Terrier	9.8	581	Y	N	6.41	4
	diab. 3	5183	14.20	M	Y	-	Miniature Poodle	6.95	unk.	N	N	14.08	42
	diab. 4	4034	11.05	F	-	Y	Mixed Breed	90	698	Y	Y	10.05	365
	diab. 5	2714	7.44	F	-	Y	Mixed Breed	17	570	N	Y	7.35	30
	diab. 6	63	0.17	M	N	-	Bernese Mountain Dog	4.46	393	N	N	0.26	0
	diab. 7	5485	15.03	F	-	Y	Boston Terrier	9.62	379	N	Y	13.23	657
	diab. 8	4772	13.07	F	-	Y	Golden Retriever	35	528	N	N	13.07	3
	diab. 9	1785	4.89	M	Y	-	Chihuahua	8	393	N	Y	4.89	0
	diab. 10	3758	10.30	M	Y	-	Yorkshire Terrier	6.64	652	?	Y	10.28	5
	diab. 11	3869	10.60	M	N	-	Mixed Breed	30.4	440	Y	N	7.57	0
	diab. 12	2955	8.10	F	-	Y	Mixed Breed	3.6	508	N	N	7.92	63
	diab. 13	4880	13.37	F	-	Y	Mixed Breed	unk.	unk.	N	N	12.88	177
	diab. 14	3343	9.16	F	-	Y	Mixed Breed	5	1106	N	N	7.55	586
	diab. 15	4393	12.04	M	Y	-	Labrador	34.1	478	N	N	unk.	unk.
	diab. 16	2720	7.45	M	Y	-	Shih Tzu	8.9	367	N	Y	6.04	516
	diab. 17	2637	7.22	M	Y	-	Dachshund	14.1	352	N	Y	7.18	18
	diab. 18	3011	8.25	M	Y	-	Pug	9.35	275	Y	N	7.02	448
	diab. 19	4234	11.60	M	N	-	Briard	33.7	473	N	N	1.89	3545
	diab. 20	4111	11.26	M	N	-	Mixed Breed	5.5	400	N	Y	11.26	0
	diab. 21	2549	6.98	F	-	Y	Labrador	22.5	773	N	N	6.98	0
	diab. 22	2405	6.59	F	-	Y	Bouvier des Flanders	38	341	N	N	3.79	1021
	diab. 23	5662	15.51	M	Y	-	Mixed Breed	8.1	345	N	N	10.51	1826
	average	3514.3	9.63	57%	69%	100%		18.5	500.1	0.2	0.3	8.2	431.3
	std error	274.3	0.75					4.2	41.1	0.1	0.1	0.8	175.9
	p vs. cont.s	0.411	0.411					0.894	< 0.0001				

Clinical characteristics of dogs included in study.

**Fig 1:**
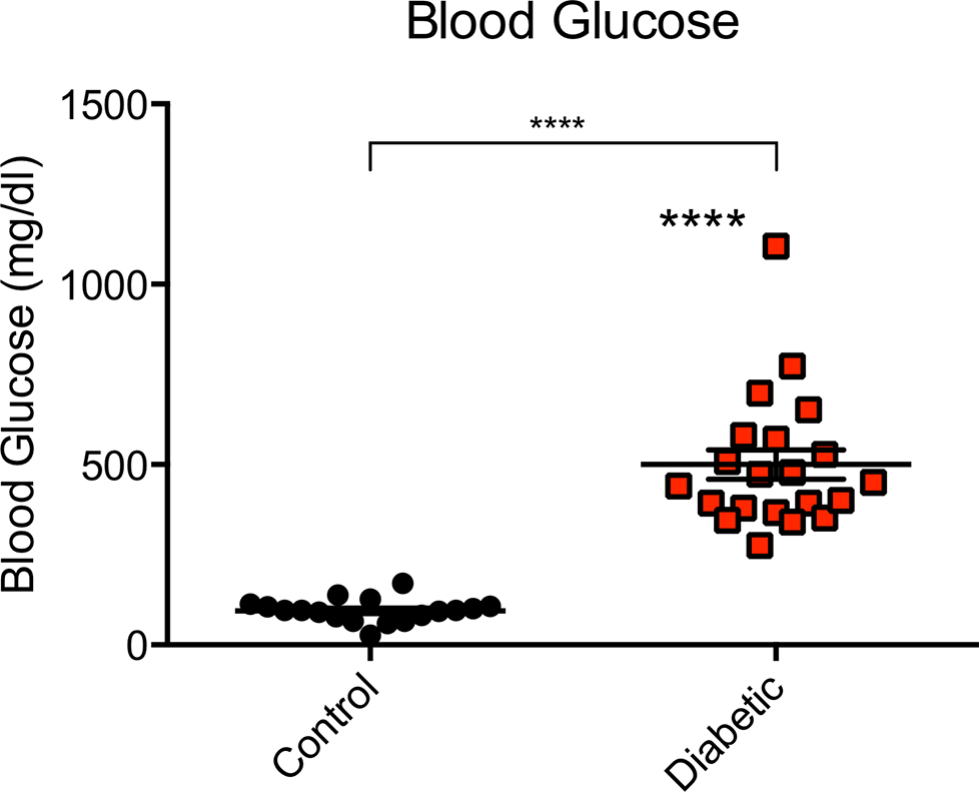
Diabetic dogs exhibit severe hyperglycemia. Blood glucose levels of control and diabetic dogs. Blood glucose in the range of 65–112 mg/dl is considered clinically normal. Results expressed as mean ± SEM for 18 control and 21 diabetic dogs. ****p<0.0001, controls vs. diabetics.

All dogs had severe diabetes prior to death. Extremely high blood glucose levels were observed, with average blood glucose of 500 mg/dl, as compared to controls with average blood glucose of 95 mg/dl (Fig 1 and Table 1). No diabetic dog had a blood glucose value of less than 275 mg/dl at the time of euthanasia. Half of the diabetic dogs presented with diabetic ketoacidosis (DKA), with serum pH less than 7.35 and urine ketones (S2 Table).

### Intact exocrine pancreata in most diabetic dogs

**Fig 2:**
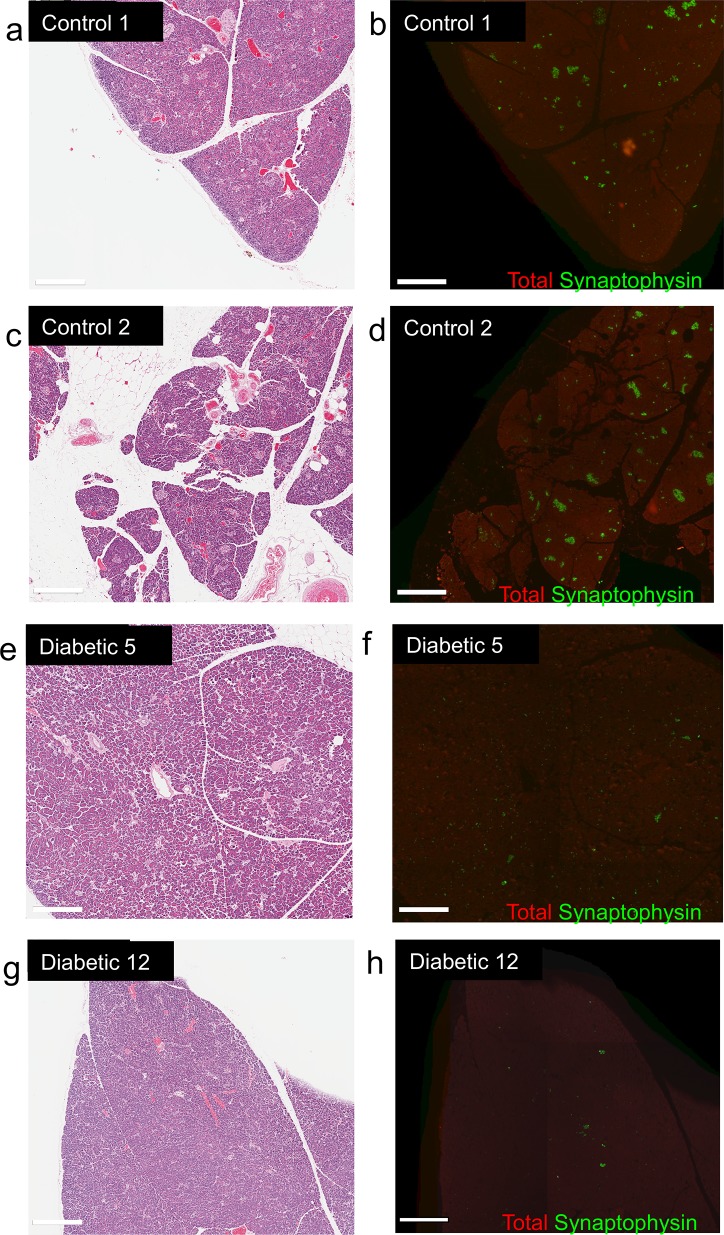
Characteristic histopathology of control and diabetic pancreata. Staining with H&E (left panels) or immunostaining (right panels) for total pancreas (red) and synaptophysin (green) of control (**a-d**) and diabetic (**e-h**) pancreata.

Pancreata were examined for histopathologic changes of the exocrine pancreas. Most dogs had no evidence of pancreatitis by H&E (Fig 2 and S1 Fig). Only 3 out of 22 diabetic dogs had any evidence of pancreatitis in H&E stained slides (S2 Table). Notably, none of the 3 cases had extensive pancreatitis: each exhibited regional histopathological changes involving a small portion of the pancreatic sample; the vast majority of exocrine pancreatic tissue was otherwise unaltered. Slides from 8 diabetic dogs and 4 controls were further stained with Masson’s Trichrome to detect fibrosis. Fibrotic changes of the pancreas are often observed in chronic canine pancreatitis [19] and in dogs with congenital diabetes [20, 21]. However, extensive fibrosis was not observed in any sample (S1 Fig and S2 Table).

### An unusual case of diabetes in a puppy

The youngest case of canine diabetes was a Bernese mountain dog at 3 months of age at diagnosis. The pancreatic histology was an obvious outlier, markedly different than other diabetic pancreata in our collection. H&E samples revealed marked, multifocal-coalescing acinar atrophy and fibroplasia (S2 Fig). Moderate diffuse neutrophil infiltration was present, as were T and B lymphocyte cells. These changes were not observed in other diabetic or control pancreata. Thus, the pathophysiology of this case of early age of onset diabetes appears to have occurred via an entirely different mechanism compared to the other cases of canine diabetes in our sample group.

### Extreme β-cell deficiency in pancreata of diabetic dogs

**Fig 3:**
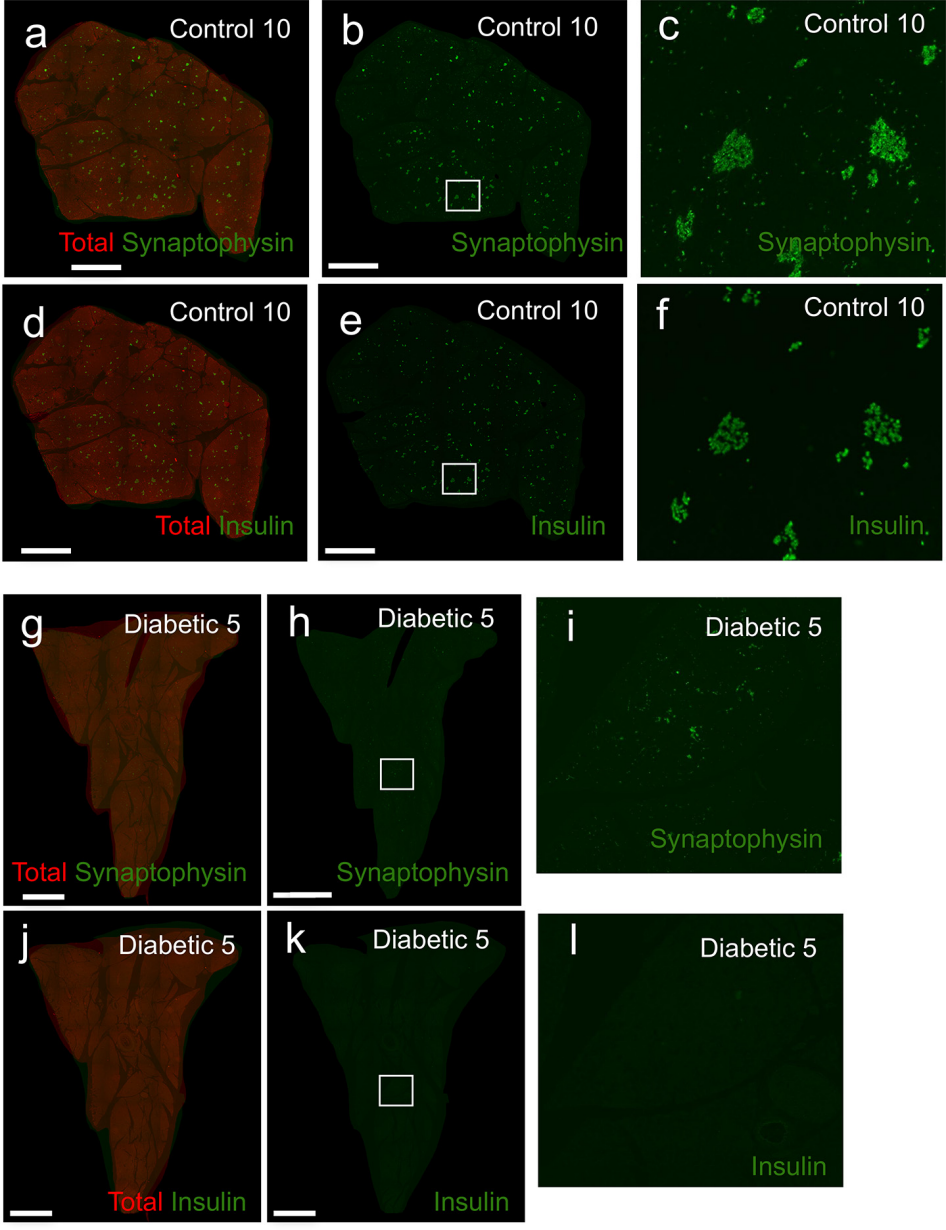
Severe islet endocrine cell and β-cell deficiency in pancreata of diabetic dogs. Representative images for control (control #10)(**a-f**) and diabetic dogs (diabetic #15)(**g-l**). Total pancreas was detected with GFP autofluorescence (red). (**a-c, g-i**) synaptophysin (green), (**d-f, j-l**) insulin (green). (**b, e, h, k**) White boxes indicate areas of interest, shown at higher magnification on right (**c, f, i, l**). Scale bars: 2mm.

A random subset of pancreatic samples were examined for histopathology of the endocrine pancreas. H&E stained slides of diabetic dogs exhibited small islets without obvious vacuolization, fibrosis, or vascular or ductal changes (Fig 2 and S1 Fig). Islets were extremely difficult to detect by H&E in diabetic pancreata. To more accurately detect islet endocrine cells and β-cells immunofluorescence imaging was performed on pancreatic samples with antisera against synaptophysin (to detect all islet endocrine cells) and insulin. The qualitative contrast between the pancreas of control and diabetic dogs was striking. Many full and intact islets were visible in the pancreas of controls (Fig 3A–3C). β-cells were also easily identifiable in most islets (Fig 3D–3F). By contrast, islets are scattered and sparse in diabetic pancreata (Fig 3G–3I). Few β-cells were observed in the diabetic pancreata (Fig 3J–3L). These observations were consistent across all diabetic sample groups (S3 and S4 Figs).

**Fig 4:**
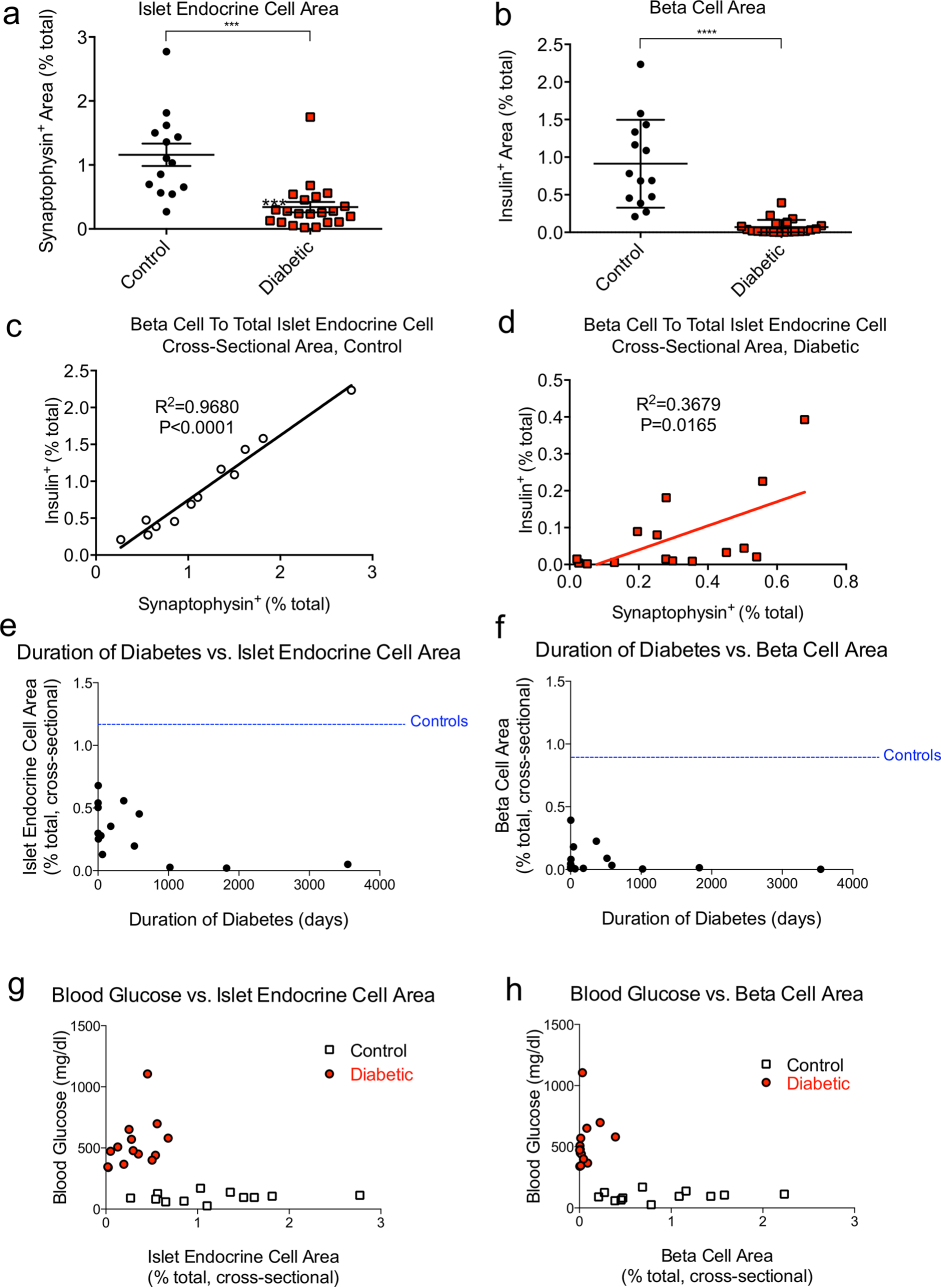
Islet endocrine cell and β-cell area are profoundly reduced in pancreata of diabetic dogs. (**a-b**) Quantification of (**a**) islet endocrine (synaptophysin^+^) cross-sectional area and (**b**) β-cell (insulin^+^) cross-sectional area, expressed as % total pancreas area per section. (**c-d**) Comparison of insulin^+^ and synaptophysin^+^ area in (**c**) control dogs and (**d**) diabetic dogs. (**a-b**) mean ± SEM for 14 control and 21 diabetic dogs. ***, p<0.001; ****, p<0.0001. Synaptophysin area and insulin area are correlated in (**c**) controls (R^2^ = 0.9680, p<0.0001) and (**d**) diabetics (R^2^ = 0.3679, p = 0.017) (**e-f**) Islet endocrine cell area and β-cell area are not correlated with duration of diabetes. Duration of diabetes vs. islet endocrine area (**e**) or β-cell area (f). Blood glucose vs. islet endocrine cell area (**g**) or β-cell area (**h**). Islet endocrine cell area or β-cell area expressed as % total pancreas area per section. Blood glucose is correlated with islet endocrine cell area (R^2^ = 0.26, P<0.05) and β-cell area (R^2^ = 0.31, P<0.05).

We carried out a detailed morphometric analysis of endocrine pancreas, acquiring low-magnification immunofluorescent images from scanned pancreata of diabetic and control samples. We quantified islet endocrine cells and β-cells with computer-aided thresholding analysis. Diabetic pancreata exhibited a nearly 3-fold decrease in cross sectional islet endocrine cell area compared to controls (Fig 4A). Cross-sectional β-cell area was even more intensely reduced in diabetics, with an approximately 12-fold decrease compared to controls (Fig 4B). We then compared the ratio of β-cell area to islet endocrine area in control and diabetes samples. β-cells were approximately 75% of the islet endocrine area in controls. However, β-cells were only 24% of the remaining total islet endocrine area in diabetics (Fig 4C).

We also interrogated the relationship in between diabetes disease duration and islet histopathology. Our sample included large cohorts of new onsets (11) and cases with longstanding diabetes (10), allowing us to test for ongoing β-cell and islet destruction. New onset and longstanding diabetes cohorts each exhibited profound reductions in islet area and β-cell area compared to controls (Fig 4E and 4F and S3 Table). Dogs with the longest standing diabetes (>1000 days) had virtually no residual islet endocrine cell area or β-cell area (Fig 4E and 4F and S3 Table). In summary, we find some evidence of ongoing destruction of islet endocrine cells and β-cells in longstanding diabetes. Notably, there was no clear relationship between islet endocrine cell cross-sectional area or β-cell cross-sectional area and blood glucose or duration of diabetes among diabetic dogs (Fig 4E–4H).

### Altered endocrine cell composition in islets of canine diabetes

**Fig 5:**
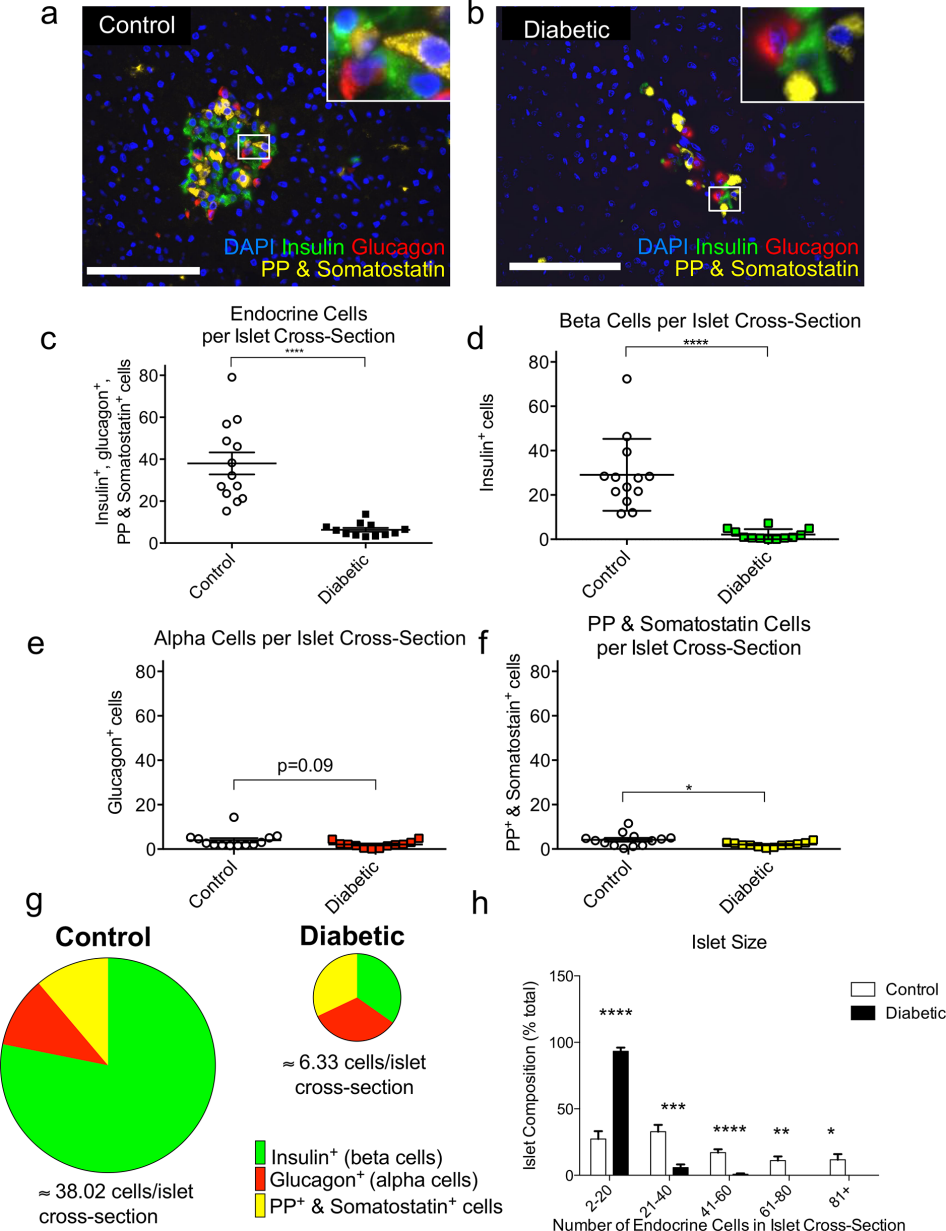
Islet size is sharply reduced and islet composition is altered in pancreata of diabetic dogs. (**a-b**) Representative pancreatic immunostaining for insulin (green), glucagon (red), PP & somatostatin (yellow), and DAPI (blue).Scale bars: 100 μm. Quantitative analysis of average number of (**c**) endocrine, (**d**) insulin, (**e**) glucagon, and (**f**) PP & somatostatin cells per islet cross-section. (**g**) Endocrine cell composition of islets. Area of each circle is proportional to the average number of endocrine cells per cross-sectional image of control (left) and diabetic (right) islets with relative fractions of insulin (green) glucagon (red), and PP & somatostatin cells (yellow). (**h**) Islet size distribution, as % of islets. All results expressed as mean ± SEM for 13 control and 12 diabetic dogs. *p<0.05; **p<0.01; ***p<0.001; ****p<0.0001.

To further understand the histopathology in diabetic dogs, we examined the morphometry of individual islets with high-resolution high-magnification (40x) fluorescent microscopy and antisera against islet hormones in a randomly chosen sample of control and diabetic pancreata. The cellular architecture of canine islets is similar to human islets in that they contain a mixture of various hormone expressing cell types throughout the center and periphery of the islet, in sharp contrast to rat and mouse islets in which β-cells are primarily in the middle of islets [22]. Islets from control pancreata were cohesive, with well-defined edges and plump islet cells (Fig 5A, S5 and S6 Figs). In contrast, diabetic islets had ill-defined boundaries and a scattered appearance of hormone expressing cells (Fig 5B, S7 and S9 Figs). The average number of islet endocrine cells was dramatically reduced in diabetic dogs, with 6-fold fewer endocrine cells per islet cross-section (Fig 5C and S4 Table). Each of the individual endocrine cell types were also reduced. β-cells per islet cross-section were hugely diminished, with more than a 13-fold decrease compared to control pancreata (Fig 5D. and S4 Table). This degree of β-cell loss closely matches our earlier calculations of β-cell cross-sectional area, as measured by with low magnification (a 5x microscope objective) analysis (Fig 5A and S4 Table). -cells were slightly decreased (P = 0.09; Fig 5E). PP and somatostatin cells were reduced by about 2-fold (Fig 5F and S4 Table). These observations were consistent across the sample groups (S4 Table).

The loss of these endocrine cell types profoundly affects the composition of the islet. In controls, β-cells made up 78% of the islet, and were centrally located in the islet (Fig 5g). 11% of the islet cells were α-cells, and the remaining 11% were PP and somatostatin containing. These results were consistent with previously published observations of non-diabetic dog islets [22]. However, in diabetics, only 30% of islet cells were β-cells, a substantial change from controls (Fig 5G). α-cells made up 40% of the islet, and PP and somatostatin cells together made up 30%. The distribution of islet size in diabetics was dramatically shifted towards smaller islets, with over 90% of diabetic cross-sectional islets containing 2–20 cells (Fig 5H). The number of endocrine cells in a cross-section of control islets was distributed more evenly, with 50% of islets containing 1–20 cells or 21–40 cells in their cross-section.

### No compensatory β-cell proliferation in diabetic dogs

We tested control and diabetic pancreata for compensatory proliferation of islet endocrine cells and β-cells. We were able to readily identify replicating cells in all of the pancreata using the proliferation marker ki67 (S10 Fig). We found zero proliferating cells within any of the 13,742 β-cells counted across 12 total control samples and 2,006 β-cells counted in diabetics (S3 Table). We counted 18,781 total islet endocrine cells across our control samples but only found 1 proliferating cell (synaptophysin+ ki67+). Similarly, we counted 10,493 total endocrine cells across our diabetic samples but only found 2 proliferating cells. Taken together, our results strongly indicate that canine diabetes is not associated with compensatory proliferation of pancreatic β-cells or islet endocrine cells.

### No insulin-glucagon co-expression in islets of diabetic dogs

Conversion from glucagon-expressing α-cells to insulin expressing β-cells has been reported under provocative conditions in mice [23]. The presence of insulin-glucagon co-expressing cells in islets is one piece of evidence cited in favor of such cell fate conversion. Indeed, insulin-glucagon co-expressing cells have been described in human pancreata from non-diabetics [24]. To test if α-cell to β-cell conversion might occur in canine diabetic pancreata we carried out an exhaustive search for islet cells that unambiguously co-expressed both insulin and glucagon. Importantly, our imaging techniques allowed us to image and quantify any possible overlap of insulin and glucagon within individual cells. We counted 15,959 endocrine cells in controls and 1,905 endocrine cells in diabetics but did not find any conclusive evidence of co-expression within control or diabetic islet cells (S4 Table). Across all of our samples only 4 cells exhibited any evidence of co-staining of insulin and glucagon. However, upon further review each of these 4 cells appeared to be α-cells that were closely adjacent to β-cells (S11 Fig). Thus, we find no direct evidence for α-cell transdifferentiation into β-cells within pancreata of diabetic dogs.

### No lymphocyte infiltration in pancreata of diabetic dogs

Infiltrating lymphocytes have rarely been detected in diabetic dogs [3, 25], which was confirmed by our studies. We selected samples to test for infiltrating lymphocytes based on availability and residual islet mass. T lymphocytes, indicated by immune-positive staining for CD3, were detected in the gut and pancreas of controls as well as diabetics (S12 Fig). 94,016 cells and 3,541 β-cells were counted in controls, and 100,589 cells and 226 β-cells were counted in diabetics (S5 Table). However, there were no CD3 positive cells near islets in any samples. Thus, islets of diabetic dogs did not exhibit evidence of ongoing infiltration by T lymphocytes.

## Discussion

Very little is known about the histopathology of canine diabetes. Our studies reveal that canine diabetes is associated with extreme β-cell loss. Diabetes in dogs led to a massive reduction of total islet endocrine cells (by ~3-fold) and β-cells (by ~13-fold). While large islets were readily found in pancreata of control dogs, islets were small and scarce in diabetic pancreata, with no evidence for pancreatitis or attempted regeneration. Confusingly, very few islet endocrine cells are present in pancreata end-stage diabetic dogs. Taken together, these results indicate that β-cell loss is a fundamental component of canine diabetes.

The cellular composition of canine islets is vastly different from human islets. A typical human islet is composed of 54% β-cells, 35% α-cells, and 11% other endocrine cells (PP, ghrelin, and somatostatin cells) [22]. We show that the insulin expressing cells represent the major component of endocrine cells in islets of healthy dogs, with approximately 80% β-cells, 10% α-cells, and 10% PP and somatostatin positive cells. Non-diabetic dog islets contain about 40 cells per cross-section. In sharp contrast, canine diabetic islets contain far fewer islet endocrine cells, about a 6-fold reduction by our estimates. However, canine diabetes is primarily associated with a profound reduction in β-cells. At clinical presentation or necropsy β-cells do not dominate any of the islets as in new onset cases of human type 1 diabetes [26]. Rather, β-cells represent just 1/3 of the islet endocrine cells due to a staggering 13.5-fold reduction compared to controls. Moreover, there was no lobar distribution of residual β-cells in diabetic dogs, as reported in human type 1 diabetes [26]. The huge reduction of β-cells during diabetes leads to very small islet size in diabetic dogs. Other islet endocrine cell types are also decreased, but to a far lesser degree. Together, these reductions leave diabetic islets with nearly equal numbers of each endocrine cell types, a radical departure from typical non-diabetic canine islets.

The late age of onset of clinical disease is an enigmatic component of canine diabetes. Whereas human type 1 diabetes often strikes young children, diabetes is typically first noted in middle aged or elderly dogs. Delays in diagnosis have been speculated to contribute to the late age of onset [3]. However, this seems exceedingly unlikely, as polydipsia and polyuria is obvious in companion animals. We speculate that intrinsic differences in the β-cell content of dog islets could influence the clinical presentation of disease. In human pancreata, the abundance of α-cells compared to β-cells could lead to a functional excess glucagon secretion compared to insulin secretion and ultimately contributes to early clinical presentation in human type 1 diabetes, as advanced by Dr. Roger Unger and others [27]. In contrast, our studies reveal that non diabetic dog islets have a much greater abundance of β-cells compared to α-cells. We speculate that this relative lack of α-cells might preserve the functional balance of insulin to glucagon after ongoing β-cell loss and thereby protect dogs from clinical presentation until late in the course of diabetes. Of course, reliable biomarkers to detect pre-diabetes in dogs will be required to fully test this hypothesis.

We saw no evidence of compensatory β-cell proliferation within islets of diabetic dogs, as previously described in human pancreata from patients with type 1 diabetes [28, 29] and in mice [30]. We found virtually no islet endocrine cell proliferation, and no β-cell proliferation in either controls or diabetics. Spontaneous islet cell proliferation has been described in younger dogs [31], and to be present “very occasionally” in islet cells of juvenile dogs less than four months of age [32]. Comparing these results to ours may be misleading, as we have previously observed that the proliferative capacity of β-cells dramatically decreases with age in mice [33]. Notably, the youngest animal that we examined for proliferation was 6.42 years old. Thus, the lack of proliferating β-cells observed in our sample may be a direct result of the advanced age of most diabetic dogs at clinical presentation.

Our samples contained no evidence of fate switching from α-cells into β-cells or vice versa. Mouse studies have described α-cells that co-express insulin and glucagon, as evidence of trans-differentiation of α-cells to a β-cells [23] or transition from β-cells to α-cells [34]. However, cells that co-express insulin and glucagon were never found in our samples from controls or diabetics. These results suggest that transformation between α and β-cells does not occur in non-diabetic or diabetic dogs. However, it remains possible that ultra-rapid fate switching might occur, without any detectable evidence of islet cells that co-express insulin and glucagon.

Pancreatitis was not an obvious feature of the pancreata in any of our canine diabetes samples. Pancreatitis has often been cited as a prominent cause of diabetes in dogs [25, 35], although the magnitude of pancreatitis as a concurrent disease has varied in different reports. In one study 24 out of 439 (11.5%) diabetic dogs were also diagnosed with pancreatitis [36]. Another study reported 8 out of 253 dogs (3.2%) with diabetes mellitus also had pancreatitis [37]. Others have reported an association of pancreatitis in diabetic dogs in 40% [38] of cases. One concern with these previously published results is the potential discrepancy between reports of pancreatitis in the medical records and histological evidence of pancreatitis. Histology is a more accurate tool for diagnosis of canine pancreatitis, as non-invasive methods are frequently not sensitive enough to detect cryptic disease [39]. We used medical records and H&E stained slides to determine whether pancreatitis was present, scanned via high-resolution digital microscopy. Although necropsy records indicated that 6 dogs of the 22 sampled had pancreatitis, in subsequent detailed analysis with the same histology tissue blocks we only found 3 dogs to truly have focal areas of pancreatitis by H&E. Therefore, pancreatitis is not the primary underlying cause of diabetes for most (if not all) of the dogs in our study.

Other potential causes of canine diabetes include cystic fibrosis-related diabetes and maturity onset diabetes of the young (MODY). Cystic fibrosis-related diabetes is an unlikely cause of canine diabetes, as cystic fibrosis was first reported to spare dogs more than 50 years ago [40]. To our knowledge this assessment remains true to this day. Additionally, we were unable to find evidence of fibrosis with Masson’s trichrome staining in 8 dogs. MODY forms of diabetes are a relatively uncommon form of diabetes, although it may represent about 1–2% of diabetes [41]. Canine diabetes might be a form of MODY diabetes, especially given the later onset of most canine diabetes cases. However, most forms of human MODY do not fit the clinical presentation of the dogs in our sample. MODY 2 is unlikely due to its typical association with kidney disease [41]; we do not observe anything similar to this in our sample population. MODY 4 is also not a probable cause, as it is usually responsible for cases with a mild form of diabetes, lacking severe hyperglycemia and is not associated with diabetic ketoacidosis [41]. MODY 1–3 and MODY 5 are still possible, but are rarely linked with DKA and are not typically associated with such extreme functional β-cell loss [41].

The youngest of diabetic samples was distinctly different from the others in pathology and seems to represent an outlier. The puppy, a Bernese mountain dog, presented with diabetes at just 3 months of age, while the next youngest dog in the study population was nearly 4 years old at onset. This very young diabetic dog exhibited evidence of lymphocytic infiltration, but not in islets, consistent with some inflammatory process, possibly a response to a viral or bacterial infection. Alternatively, the dog could have succumbed to some sort of pancreatic autoimmune process. Regardless, the puppy seems to have had a distinct form of diabetes. Very early age of onset canine diabetes cases have been occasionally described, and are estimated to be about 1.5% of all diabetic cases [42]. Neonatal diabetes has been described in Labrador retrievers, which are closely related to Bernese mountain dogs [43]. In this respect our case of neonatal diabetes is quite similar to those previously reported, although the underlying cause remains a mystery. Additional studies on this rare form of canine diabetes are clearly warranted.

We find no direct evidence for autoimmunity in pancreata of dogs with diabetes. Although autoimmunity has been often cited as a possible mechanism underlying β-cell loss in canine diabetes, direct evidence supporting this hypothesis is sparse. About half of new onset cases in one study had anti-insulin antibodies [44]. Antibodies against the self-antigens Gad65 and IA2 were detected in a minority (5 of 30) of dogs with newly diagnosed diabetes [13]. However, a comprehensive study recently reported lack of evidence of islet autoimmunity in sera from 121 diabetic dogs against pancreata of 133 healthy dogs [10]. Indeed, to our knowledge infiltrating lymphocytes have only been detected in very young diabetic dogs [7]. We found no islet infiltration (as measured by the presence of CD3+ cells) in 6 diabetic dogs. A few rare CD3+ cells were detected in the acinar tissue of the pancreas of 50% of the controls and 50% of the diabetics. The lack of lymphocytic infiltration in our samples is inconsistent with an acute islet autoimmune process. However, such lymphocytic infiltration is also rare in human Type 1 diabetes [45]. A new view of human autoimmune diabetes has emerged where β-cell destruction is far slower than previously appreciated. Notably, the islets of control dogs are largely comprised of β-cells. As a result, β-cell destruction reduces the vast majority of the cellular contents of the islets, leaving only a few random α, β, PP, and ghrelin cells. This histologic appearance is completely different from that of type 1 human diabetes [46]. This distinction allows for a better understanding of canine diabetes as a devastating reduction of β-cells, diminishing the dominant representation of β-cells in islets. Together, these findings hint that diabetes in dogs results from autoimmune destruction of β-cells. Studies to detect infiltrating lymphocytes within islets of pre-diabetic dogs will be essential to confirm autoimmunity as a cause of canine diabetes.

In summary, our results advance the field by providing a precise quantitative analysis of islets within the pancreata of diabetic dogs. At previous studies have reported a qualitative loss of islets and β-cells in diabetic dogs [7, 17]. However, there is a notable gap in our knowledge of pancreas and islet morphometry within diabetic dogs. We hope that our results inform future studies to derive better therapies for this common and destructive disease of companion animals, and by extension advance therapies for human type 1 diabetes.

## Supporting Information

S1 Table Cause of death and underlying diagnosis for control and diabetic dogs. (XLSX)

S2 Table Clinical characteristics of diabetic ketoacidosis (DKA) or pancreatitis. (**a**) Clinical evaluation of DKA. Venous blood gas from serum of diabetic dogs. DKA defined as diabetic (hyperglycemia) with pH below 7.3 and ketones in urine. (**b**) Clinical and histological evaluation of pancreatitis from medical records and, definitively, from H&E staining (see S2A–S2I Fig). (**c**) Histological evaluation of fibrosis from slides with Masson’s Trichrome staining (see S2J–S2L Fig).
(XLSX)

S3 Table Proliferating endocrine cells are rare in controls and diabetics. Individual data from control or diabetic dogs detailing area and proliferation results. (**a-c**) Morphometry analysis of control and diabetic pancreata. (**d-e**) β-cell proliferation analysis of control and diabetic pancreata. Control: 0 ki67^+^ insulin^+^ cells of 13,742 insulin^+^ cells. Diabetic: 0 ki67^+^ insulin^+^ of 2,006 insulin^+^ cells. (**f-g**) Islet endocrine cell proliferation analysis in controls and diabetics. Control: 1 ki67+ synaptophysin+ cell of 18,781 synaptophysin+ cells. Diabetic: 1 ki67+ synaptophysin+ cells of 10,493 synaptophysin+ cells.
(XLSX)

S4 Table Insulin-glucagon co-expression was never found in any pancreata of control or diabetic dogs. Individual data from control or diabetic dogs detailing islet composition and analysis of insulin-glucagon co-expression.(a) Islet composition and size are considerably impacted by diabetes. (b) Analysis of insulin-glucagon co-expression. Control: 0 insulin+ glucagon+ cells of 15,959 endocrine cells. Diabetic: 0 insulin+ glucagon+ cells of 1,905 endocrine cells.
(XLSX)

S5 Table CD3^+^ cells are detected in gut and pancreas, but not found to infiltrate islets. Neither diabetic dogs nor control dogs had pancreatic islets with infiltrating CD3^+^ cells. Quantification and analysis of CD3^+^ cells in control and diabetic pancreata. Control: 14 CD3^+^ cells of 94,016 DAPI^+^ cells. Diabetic: 26 CD3^+^ cells of 100,589 DAPI^+^ cells.
(XLSX)

S1 Fig Images of pancreatic sections stained with H&E or Masson’s Trichrome stain. Low power (**a, d, g**), high power (**b, e, h**), and highest power (**c, e, i**) views of H&E staining of Diabetic 3, without pancreatitis (**a-c**), Diabetic 15, with pancreatitis from medical records but without pancreatitis from H&E staining (**d-f**), and Diabetic 10, with pancreatitis (**g-i**). Scale bars: 2 mm in low and high power views, 0.5mm in highest power view. Low power (**j, m**), high power (**k, n**), and highest power (**l, o**) views of Masson’s Trichrome staining of Control 10, without fibrosis (**j-l**), and Diabetic 22, without fibrosis (**m-o**).
(JPG)

S2 Fig Histological analysis of the youngest dog in study reveals likely infectious etiology of diabetes. Pancreas of young dog, Diabetic 6, stained with hematoxylin and eosin (**a**) Low power view of pancreas. (**b**) High power view of pancreas. (**c**) Highest power view of pancreas, revealing neutrophil and lymphoplasmacytic inflammation. Scale bars: 2 mm in low and high power views, 0.5mm in highest power view.
(JPG)

S3 Fig Histopathology of pancreas of control dogs. Representative images for control dogs. Total pancreas was detected with autofluorescence (red). (**a-c, g-i**) synaptophysin (green), (**d-f, j-l,**) insulin (green). (**b, e, h, k**) White boxes indicate areas of interest, shown at higher magnification on right (**c, f, i, l,**). Scale bars: 2 mm.
(JPG)

S4 Fig Histopathology of pancreata of diabetic dogs shows consistently minimal islet endocrine and β-cell area. Representative images for diabetic dogs. Total pancreas was detected with autofluorescence (red). (**a-c, g-i**) synaptophysin (green), (**d-f, j-l,**) insulin (green). (**b, e, h, k**) White boxes indicate areas of interest, shown at higher magnification on right (**c, f, i, l**). Scale bars: 2 mm.
(JPG)

S5 Fig Histopathology of islets from pancreata of control dogs. Staining with H&E (**a-d**) or immunostaining (**e-h**) for insulin (green), glucagon (red), PP & Somatostatin (yellow) and DAPI (blue) of control pancreata. Scale bars: 100 μm.
(JPG)

S6 Fig Histopathology of islets from pancreata of control dogs. Staining with H&E (**a-b**) or immunostaining (**c-d**) for insulin (green), glucagon (red), PP & Somatostatin (yellow) and DAPI (blue) of control pancreata. Scale bars: 100 μm.
(JPG)

S7 Fig Histopathology of islets from pancreata of diabetic dogs. Staining with H&E (**a-d**) or immunostaining (**e-h**) for insulin (green), glucagon (red), PP & Somatostatin (yellow) and DAPI (blue) of diabetic pancreata. Scale bars: 100 μm.
(JPG)

S8 Fig Histopathology of islets from pancreata of diabetic dogs. Staining with H&E (**a-d**) or immunostaining (**e-h**) for insulin (green), glucagon (red), PP & Somatostatin (yellow) and DAPI (blue) of diabetic pancreata. Scale bars: 100 μm.
(JPG)

S9 Fig Histopathology of islets from pancreata of diabetic dogs. Staining with H&E (**a**) or immunostaining (**b**) for insulin (green), glucagon (red), PP & Somatostatin (yellow) and DAPI (blue) of diabetic pancreata. Scale bars: 100 μm.
(JPG)

S10 Fig Proliferating endocrine cells are rarely found in controls or diabetics. Rare non-representative pictures of pancreata of control and diabetic dogs stained to detect proliferation. Immunostaining for DAPI (blue), synaptophysin (yellow), insulin (green), ki67 (red).(**a**) Proliferating endocrine cell in a control (**b**) Intra-islet (non-endocrine) proliferation in a control. (**c**) Non-endocrine proliferating cell in close proximity to an islet in a control. (**d**) Proliferating endocrine cell in a diabetic (**e**) Non-endocrine proliferating cell in close proximity to an islet in a diabetic. Scale bars: 100 μm.
(JPG)

S11 Fig Insulin-glucagon co-expression was never found in any pancreata of control or diabetic dogs. Immunostaining for DAPI (blue), insulin (green), and glucagon (red). Rare, non-representative images of closest possible proximity in between insulin and glucagon illustrating that distinct insulin and glucagon expressing cells can be intimately related but without co-expression of both hormones within the same cell. (**a-b**) controls and (**c-d**) diabetics. Scale bars: 25 μm.
(JPG)

S12 Fig Islets with infiltrating CD3+ cells were only found in pancreas of the youngest dog. Immunostaining for CD3 (green), insulin (red), and DAPI (blue) in (**a-b**) control gut, (**c-d**) control pancreas, (**e-f**) diabetic gut, and (**g-h**) diabetic pancreas. (**i-j**) Youngest dog in study (4 months of age), Diabetic 6, had vast number of lymphocytes present in exocrine pancreas. Scale bars: 100 μm.
(JPG)
