# Supplementary figures and images for: Extreme Beta-Cell Deficiency in Pancreata of Dogs with Canine Diabetes
Source: PLoS One. 2015 Jun 9;10(6):e0129809. doi: 10.1371/journal.pone.0129809 (PMC4461304; doi:10.1371/journal.pone.0129809)

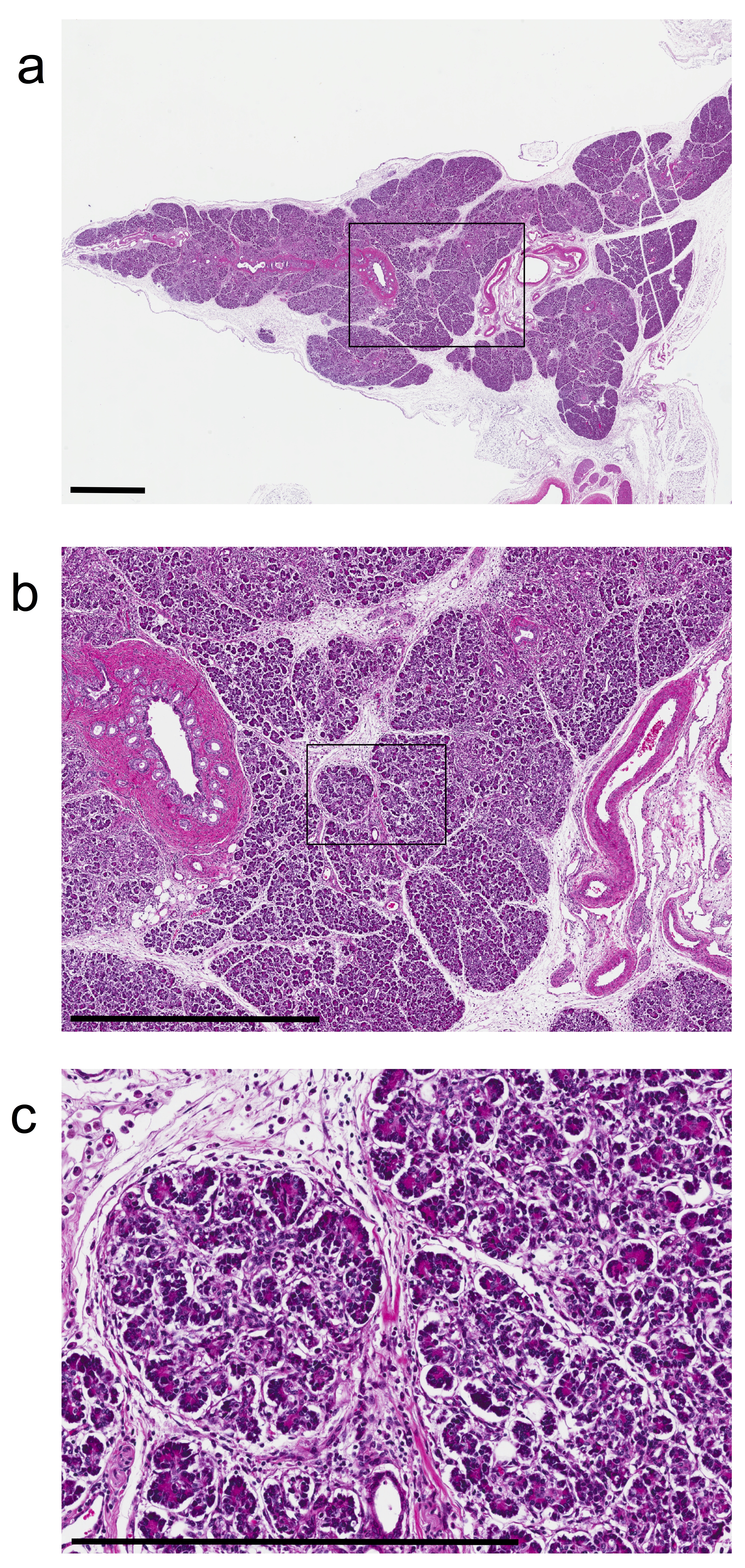

Supplement: S2 Fig — Pancreas of young dog, Diabetic 6, stained with hematoxylin and eosin (a) Low power view of pancreas. (b) High power view of pancreas. (c) Highest power view of pancreas, revealing neutrophil and lymphoplasmacytic inflammation. Scale bars: 2 mm in low and high power views, 0.5mm in highest power view. (JPG) [file pone.0129809.s007.jpg]

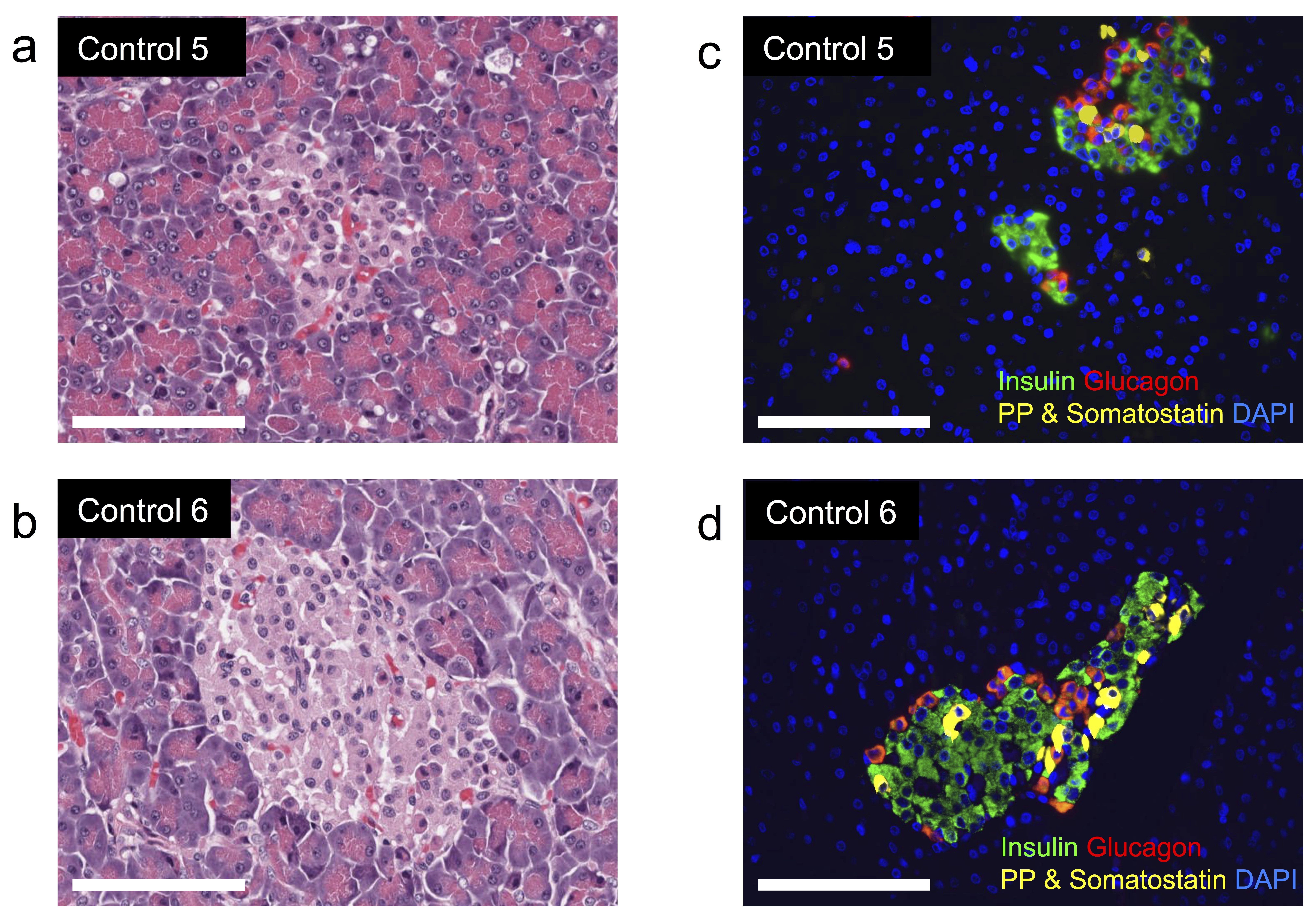

Supplement: S6 Fig — Staining with H&E (a-b) or immunostaining (c-d) for insulin (green), glucagon (red), PP & Somatostatin (yellow) and DAPI (blue) of control pancreata. Scale bars: 100 μm. (JPG) [file pone.0129809.s011.jpg]

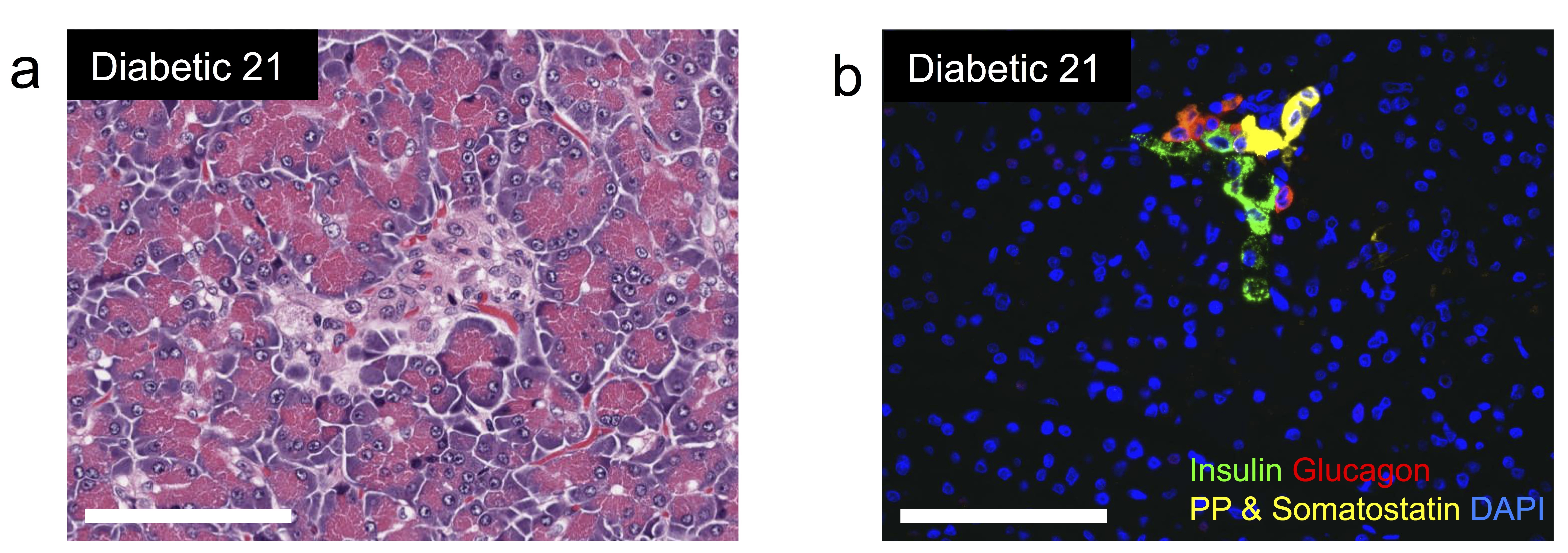

Supplement: S9 Fig — Staining with H&E (a) or immunostaining (b) for insulin (green), glucagon (red), PP & Somatostatin (yellow) and DAPI (blue) of diabetic pancreata. Scale bars: 100 μm. (JPG) [file pone.0129809.s014.jpg]

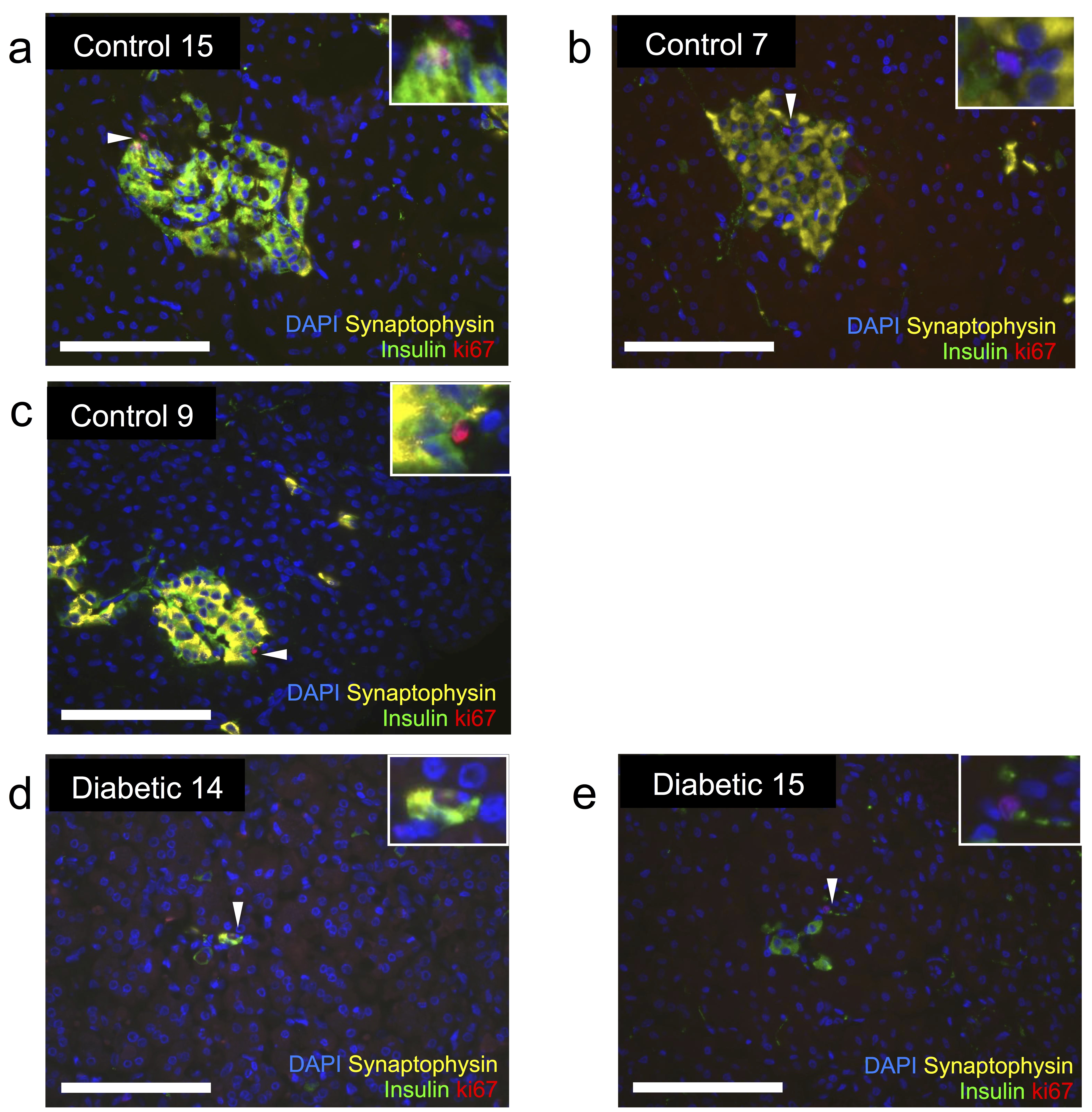

Supplement: S10 Fig — Rare non-representative pictures of pancreata of control and diabetic dogs stained to detect proliferation. Immunostaining for DAPI (blue), synaptophysin (yellow), insulin (green), ki67 (red).(a) Proliferating endocrine cell in a control (b) Intra-islet (non-endocrine) proliferation in a control. (c) Non-endocrine proliferating cell in close proximity to an islet in a control. (d) Proliferating endocrine cell in a diabetic (e) Non-endocrine proliferating cell in close proximity to an islet in a diabetic. Scale bars: 100 μm. (JPG) [file pone.0129809.s015.jpg]

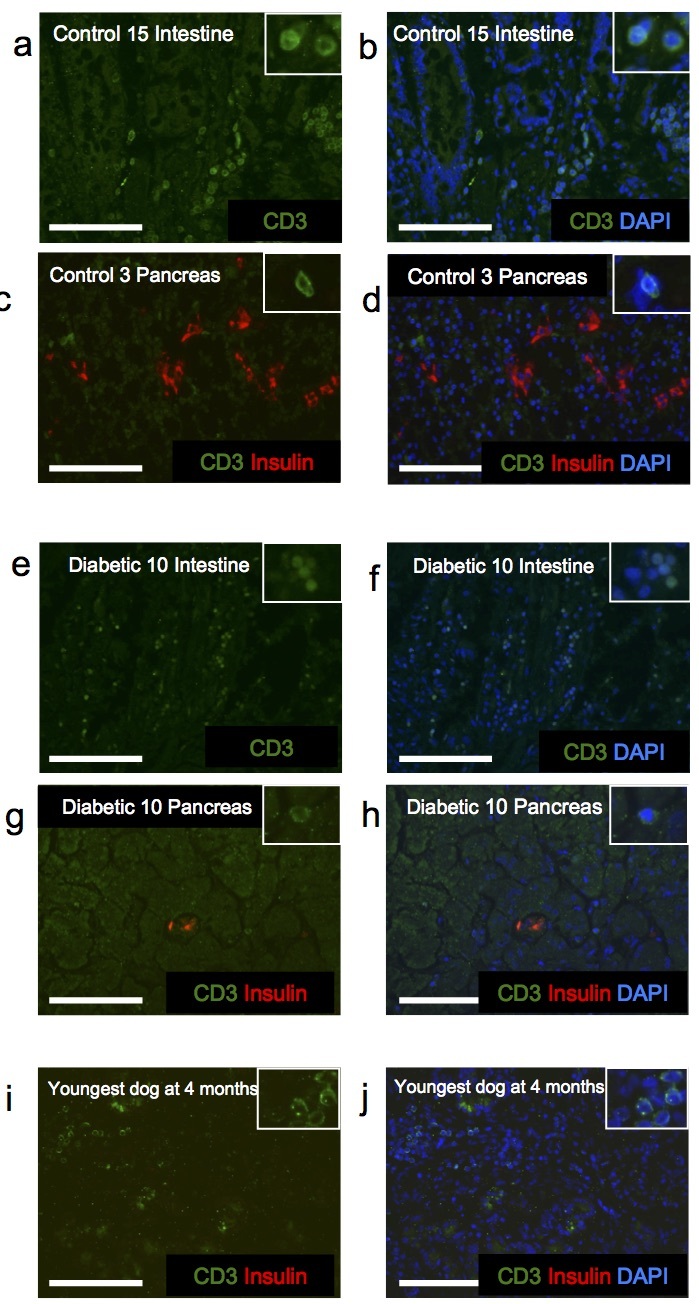

Supplement: S12 Fig — Immunostaining for CD3 (green), insulin (red), and DAPI (blue) in (a-b) control gut, (c-d) control pancreas, (e-f) diabetic gut, and (g-h) diabetic pancreas. (i-j) Youngest dog in study (4 months of age), Diabetic 6, had vast number of lymphocytes present in exocrine pancreas. Scale bars: 100 μm. (JPG) [file pone.0129809.s017.jpg]
